# Impact of Age-Friendliness on Older Adults’ Consumption and Well-Being: Insights from a Hong Kong Survey Experiment

**DOI:** 10.1093/geroni/igaf122.527

**Published:** 2025-12-31

**Authors:** Xue Bai, Mengyu Liu, Youjuan Zhang, Shuai Zhou

**Affiliations:** The Hong Kong Polytechnic University, Hong Kong, China (People’s Republic); The Hong Kong Polytechnic University, Hong Kong, China (People’s Republic); The Hong Kong Polytechnic University, Hong Kong, China (People’s Republic); The Hong Kong Polytechnic University, Hong Kong, China (People’s Republic)

## Abstract

Aims This study aims to investigate how older adults in Hong Kong perceive age-friendliness, focusing on how age-friendly business/societal structures influence consumption choices and wellbeing. Methods We use the second wave data from the Panel Study on Active Ageing and Society (PAAS, N = 3000), which is the first city-wide representative longitudinal survey on active ageing and society in Hong Kong. To identify the causal effect of age-friendliness, we designed a factorial survey experiment and randomly assign respondents to 16 self-developed vignettes. Multilevel models were employed to analyse the data. Results Our findings show that age-friendly practices, in general, along with its four specific domains of physical environment, staff and personnel, marketing and information, and products and services, positively impacts the consumption willingness of adults aged≥50 years, increasing their willingness to consume by 146% (mean difference: 4.03, t = 32.07, P < 0.001). Additionally, satisfaction with AFB is positively associated with the physical (e.g. lower risk of falls, sarcopenia and frailty), mental (lower risk of depression and loneliness), and cognitive health of older adults, as well as their life satisfaction. Businesses perceived as age-friendly are more likely to motivate older adults to engage in consumption that aligns with healthy ageing goals. Conclusions Age-friendliness enhances healthy aging by shaping consumption behaviors that prioritize older adults’ holistic well-being. The findings highlight actionable strategies for businesses and policymakers to design inclusive markets and age-friendly cities. By addressing older adults’ expectations of the silver market, this study supports creating health-supportive environments where older adults thrive.

